# Recanalization Treatments for Pediatric Acute Ischemic Stroke in France

**DOI:** 10.1001/jamanetworkopen.2022.31343

**Published:** 2022-09-15

**Authors:** Manoëlle Kossorotoff, Basile Kerleroux, Grégoire Boulouis, Béatrice Husson, Kim Tran Dong, François Eugene, Lena Damaj, Augustin Ozanne, Céline Bellesme, Anne Rolland, Romain Bourcier, Aude Triquenot-Bagan, Gaultier Marnat, Jean-Philippe Neau, Sylvie Joriot, Alexandra Perez, Maud Guillen, Maximilien Perivier, Frederique Audic, Jean François Hak, Christian Denier, Olivier Naggara

**Affiliations:** 1 Assistance publique-Hôpitaux de Paris, French Center for Pediatric Stroke, France; 2 Pediatric Neurology Department, Assistance publique-Hôpitaux de Paris, Inserm, Hôpital Necker-Enfants malades, Paris, France; 3 Pediatric Radiology Department, Assistance publique-Hôpitaux de Paris, Hôpital Necker-Enfants malades, Paris, France; 4 GHU Paris Psychiatrie et Neurosciences, CH Sainte-Anne, Inserm, Université de Paris Cité, Institut de psychiatrie et neurosciences de Paris, Service d'imagerie morphologique et fonctionnelle, UMRS1266, Paris, France; 5 Neuroradiology, Tours University, CHRU Bretonneau, Tours, France; 6 Pediatric Radiology Department, Assistance publique-Hôpitaux de Paris, Université de Paris-Saclay, Hôpital Bicêtre, le Kremlin-Bicêtre, France; 7 Neuroradiology, Rennes University, CHU de Rennes, Rennes, France; 8 Pediatric Department, Rennes University, CHU de Rennes, Rennes, France; 9 Department of Interventional Neuroradiology Neuro Brain Vascular Center, Assistance publique-Hôpitaux de Paris, Paris-Saclay University, Hôpital Bicêtre, le Kremlin-Bicêtre, France; 10 Pediatric Stroke Unit and Pediatric Neurology Department, Assistance publique-Hôpitaux de Paris, Paris-Saclay University, Hôpital Bicêtre, le Kremlin-Bicêtre, France; 11 Neurology Department, Nantes University, CHU de Nantes, Nantes, France; 12 Pediatric Department, Nantes University, CHU de Nantes, Nantes, France; 13 Neurology Department, Rouen University, CHU de Rouen, Rouen, France; 14 Neuroradiology Department, Bordeaux University, CHU de Bordeaux, Bordeaux, France; 15 Neurology Department. Poiters University, CHU de Poitiers, Poitiers, France; 16 Pediatric Neurology Department, Lille University, CHU de Lille, Lille, France; 17 Pediatric Department, Strasbourg University, CHU de Strasbourg, Strasbourg, France; 18 Neurology Department, Rennes University, CHU de Rennes, Rennes, France; 19 Pediatric Department, Tours University, CHU de Tours, Tours, France; 20 Pediatric Neurology Department, Aix-Marseille University, CHU la Timone, Marseille, France; 21 Neuroradiology, Aix-Marseille University, CHU la Timone, Marseille, France

## Abstract

**Importance:**

There is to date limited evidence that revascularization strategies are associated with improved functional outcome in children with acute ischemic stroke (AIS).

**Objectives:**

To report clinical outcomes and provide estimates of revascularization strategy safety and efficacy profiles of intravenous thrombolysis (IVT) and/or endovascular treatment (EVT) in children with AIS.

**Design, Setting, and Participants:**

The KidClot multicenter nationwide cohort study retrospectively collected data of children (neonates excluded) with AIS and recanalization treatment between January 1, 2015, and May 31, 2018. Data analysis was performed from January 1, 2015, to May 31, 2019.

**Exposure:**

IVT and/or EVT.

**Main Outcomes and Measures:**

Primary outcome was day 90 favorable outcome (modified Rankin Scale [mRs] 0-2, with 0 indicating no symptoms and 6 indicating death). Secondary end points included 1-year favorable outcome (mRs, 0-2), mortality, and symptomatic intracerebral hemorrhage. Other measures included the Pediatric National Institutes of Health Stroke Scale (pedNIHSS), with pedNIHSS 0 indicating no symptoms, 1 to 4 corresponding to a minor stroke, 5 to 15 corresponding to a mild stroke, greater than 15 to 20: severe stroke, and the adult Alberta Stroke Program Early CT Score (ASPECTS), which provides segmental assessment of the vascular territory, with 1 point deducted from the initial score of 10 for every region involved (from 10 [no lesion] to 0 [maximum lesions]).

**Results:**

Overall, 68 children were included in 30 centers (IVT [n = 44]; EVT [n = 40]; 44 boys [64.7%]; median [IQR] age, 11 [4-16] years; anterior circulation involvement, 57 [83.8%]). Median (IQR) pedNIHSS score at admission was 13 (7-19), higher in the EVT group at 16 (IQR, 10-20) vs 9 (6-17) in the IVT only group (*P* < .01). Median time from stroke onset to imaging was higher in the EVT group at 3 hours and 7 minutes (IQR, 2 hours and 3 minutes to 6 hours and 24 minutes) vs 2 hours and 39 minutes (IQR, 1 hour and 51 minutes to 4 hours and 13 minutes) (*P* = .04). Median admission ASPECTS score was 8 (IQR, 6-9). The main stroke etiologies were cardioembolic (21 [30.9%]) and focal cerebral arteriopathy (17 [25.0%]). Median (IQR) time from stroke onset to IVT was 3 hours and 30 minutes (IQR, 2 hours and 33 minutes to 4 hours and 28 minutes). In the EVT group, the rate of postprocedure successful reperfusion (≥modified Treatment in Cerebral Infarction 2b) was 80.0% (32 of 40). Persistent proximal arterial stenosis was more frequent in focal cerebral arteriopathy (*P* < .01). Death occurred in 3 patients (4.4%). Median pedNIHSS reduction at 24 hours was 4 (IQR, 0-9) points. Intracerebral hemorrhage occurred in 4 patients and symptomatic intracerebral hemorrhage occurred in 1 patient, all in the EVT group. The median mRS was 2 (IQR, 0-3) at day 90 and 1 (IQR, 0-2) at 1 year, which was not significantly different between EVT and IVT only groups, although different in initial severity.

**Conclusions and Relevance:**

The findings of this cohort study suggest that use of EVT and/or IVT is safe in children with AIS.

## Key Points

**Question:**

In children with acute ischemic stroke, are intravenous thrombolysis (IVT) and/or endovascular treatment (EVT) safe and effective revascularization strategies?

**Findings:**

In this cohort study of 68 children treated with IVT (n = 44) and/or EVT (n = 40), favorable neurologic outcome was observed in 78% at 1 year. Complication rates of revascularization treatments were low and main outcomes were not significantly different between the EVT and no-EVT groups, although the condition of patients who received EVT was initially more severe.

**Meaning:**

The findings of this cohort study suggest that use of IVT and/or EVT may be safe in children with acute ischemic stroke.

## Teaser

This cohort study compares the use of intravenous thrombolysis with endovascular treatment in children with acute ischemic stroke.

## Introduction

The incidence of acute arterial ischemic stroke (AIS) is known to increase with age, but AIS is not uncommon in children.^1^ Acute arterial ischemic stroke during childhood, with an estimated incidence of 2 to 8 per 100 000 children-year, is an important burden of acquired disability and has long-term socioeconomic and psychosocial outcomes.^2^

The field of AIS has seen major evolutions in adults in the past decades with the advent of intravenous recombinant tissue plasminogen activator (r-tPA) treatment and endovascular treatment (EVT) for select patients older than 18 years^3^; however, to our knowledge, there is limited evidence that revascularization strategies are associated with improved functional outcome in children.^4^ In turn, intravenous r-tPA and EVT are administered to children on a case-by-case basis, using indirect evidence derived from adult clinical trials. Beyond the undisputed pathophysiologic rationale of the benefits of blood flow restoration to salvageable hypoperfused brain tissue in the context of acute brain arterial occlusion,^5,6^ many questions on the applicability, safety, and benefits of brain revascularization strategies in children remain.^5^ Differences in stroke etiologies, coagulation system maturation, rarity of the condition leading to delayed diagnosis, and lack of experience may limit the applicability of treatments with proven benefit in adult stroke to pediatric stroke. Reports of intravenous thrombolysis (IVT) in children suggested a positive safety profile, despite the limited number of patients included.^7,8^ The SaveChildS study^9^ analyzed data from a multicenter cohort that included 73 children treated with EVT between 2000 and 2018. The investigators reported that EVT appeared to have a positive safety profile in children with large-vessel occlusion, similar to the results of the largest real-world prospective adult registries, and that long-term neurologic outcome was good in most patients. However, the generalizability of these results remains questionable because of the overrepresentation of cardioembolic events in this study and the risk of selection bias over a 220-month inclusion period in 27 centers. Recently, Dicpinigaitis et al^10^extracted data on a cohort of 190 children treated with EVT from the US National Inpatient Sample and supported the safety profile of EVT in a large pediatric sample. However, the generalizability of results remains limited by many approximations regarding the population characteristics and outcomes owing to the lack of key variables, such as the National Institutes of Health Stroke Scale (NIHSS) score or modified Rankin Scale score. At times of profound modifications in AIS treatment paradigms, attempts at optimizing systems of care toward pediatric stroke-ready networks have shown interest,^7^ and both technical advances and better understanding of underlying etiologies of pediatric stroke have positioned the issue of pediatric AIS acute revascularization in the limelight. In a national multicenter retrospective study of consecutive children with AIS treated with IVT (using r-tPA) and/or EVT we aimed to provide an up-to-date evaluation of revascularization strategies in the pediatric population.

## Methods

### Study Design and Patient Selection

The National Retrospective Study of Recanalization Treatments in Pediatric Arterial Ischemic Stroke (KID-CLOT) is a multisource, retrospective study across France collecting data on consecutive children aged 28 days to 18 years with acute AIS who received a recanalization treatment and registered on ClinicalTrials.gov (NCT03887143).

This retrospective analysis received approval from the ethics committee of the French National Pediatrics Society. Patients and/or legal guardians were informed and gave oral consent for the children to be included in this study; no financial compensation was provided. This study followed the Strengthening the Reporting of Observational Studies in Epidemiology (STROBE) reporting guideline.

Patients were included in the study if they met the following criteria: (1) age 28 days to 18 years at the time of stroke onset, (2) radiologically confirmed acute AID (ie, patient had both radiographic evidence of an infarct in an arterial distribution and consistent neurologic signs and symptoms), (3) received IVT and/or EVT and/or digital subtraction arteriography in an intention-to-treat protocol with EVT at the acute phase of AIS, (4) residence in mainland France or overseas French territories, and (5) presented during the inclusion period(January 1, 2015, to May 31, 2018).

### Recruiting Centers and Validation Method

We hypothesized that care for every child with AIS receiving a recanalization treatment would be managed at a tertiary care center. Thus, stroke centers, pediatric neurology departments, and interventional neuroradiology departments were separately contacted at every academic center in France and asked whether they had treated at least 1 pediatric patient during the study period. Duplicates across departments were identified and resolved. The Paris-Ile-de-France region, the most populated area of France, comprising Paris, served as a validation region for this method. In this region, every stroke unit was contacted, including nonacademic centers, to assess whether patients may be overlooked by focusing on academic centers.

### Data Acquisition

Local investigators collected information on clinical and biological parameters at admission, including demographic data (age, sex), stroke presentation, clinical and biological assessment (including pediatric NIHSS [pedNIHSS] score, blood pressure value, coagulation parameters, and serum glucose level before treatment), and medical history and prestroke level of independence. The coordinating investigator (M.K.) centrally reviewed all score ratings, especially if the ratings were inferred from medical records.^11,12^

A panel of 3 neuroradiologists (B.K., B.H., and O.N.) blinded to clinical data centrally reviewed initial, procedural, and follow-up imaging data: brain computed tomography (CT) and/or magnetic resonance imaging (MRI), CT or MR angiography, digital subtraction angiography, and posttreatment brain CT and/or MRI. The neuroradiologists rated the extent of the infarct in 2 ways. First, the infarct was rated according to the adult Alberta Stroke Program Early CT Score (ASPECTS) or posterior circulation ASPECTS depending on the vascular territory involved on initial CT and/or MRI. ASPECTS provides segmental assessment of the vascular territory, and 1 point is deducted from the initial score of 10 for every region involved (from 10 [no lesion] to 0 [maximum lesions]).^13^ Second, the infarct was rated according to the ABC/2 method, which consists of 3 orthogonal linear measures to estimate the core volume.^14^

### Stroke Etiology

Parameters of baseline stroke workup were collected, including cardiac ultrasonography, CT and/or MRI, and CT angiography or MR angiography of the head and neck, and screening for infection, metabolic disease, coagulation abnormality, and vasculitis.^15^ For each patient, a centrally performed multidisciplinary symposium determined stroke etiology according to the Childhood Arterial Ischemic Stroke Standardized Classification and Diagnostic Evaluation Classification measures,^15^ which distinguishes the following subtypes: (1) unilateral or bilateral focal cerebral arteriopathy (FCA), (2) cardioembolic, (3) cervical artery dissection, (4) thrombotic, (5) other etiologies, and (6) multifactorial or unknown.

### Recanalization Treatments and Pathway of Care

In this retrospective study, the final decision on treatment eligibility was at the discretion of the multidisciplinary team at included sites. Criteria for intravenous r-tPA and/or EVT eligibility in adults (eg, time from symptom onset, NIHSS score, absence of contraindication, and ASPECT score) were retrieved to estimate a posteriori the degree of adherence to and transposition from practice guidelines in adults.

We recorded the following variables when applicable: time metrics, including time from symptom onset (or last-seen well) to hospital presentation to first imaging to first recanalization treatment and, when appropriate, to groin puncture and recanalization, and technical characteristics of recanalization treatments (infusion characteristics, initial occlusion location on digital subtraction arteriography, EVT technical strategy, devices used, and number of passes performed during EVT). Stroke pathway-of-care data were collected, including hospital-to-hospital transfers and management in a pediatric or adult ward.

### Outcome Definitions

Patients treated with EVT had an immediate posttreatment recanalization assessment according to the extended modified Treatment in Cerebral Infarction (mTICI) score (mTICI 0 indicates no perfusion; grade 1, antegrade reperfusion past the initial occlusion but limited distal branch filling; grade 2a, antegrade reperfusion of less than half of the occluded target artery previously ischemic territory; grade 2b, antegrade reperfusion of more than half of the previously occluded target artery ischemic territory; grade 2c, near-complete perfusion except for slow flow in a few distal cortical vessels or presence of small distal cortical emboli; and grade 3, complete antegrade reperfusion, with an absence of visualized occlusion in all distal branches). A TICI score greater than or equal to 2b was considered to indicate successful recanalization.^16^ Early clinical outcome was evaluated for all patients using the pedNIHSS at 24 hours and day 7 after admission and the modified Rankin Scale (mRS) at hospital discharge (from 0 [no symptom], to 6 [death]).

Clinical and radiologic complications related to stroke and/or stroke treatment were recorded: malignant cerebral artery infarct, hemorrhagic transformation, or stroke-related mortality. Symptomatic intracerebral hemorrhagic transformation was defined according to the ECASS-II definition^17^ as any intracranial hemorrhage on imaging control performed 24 hours after mechanical thrombectomy that was associated with neurologic deterioration (increase of ≥4 points in the pedNIHSS score from baseline).

Long-term outcome was evaluated using mRS at 90 days and 12-month follow-up and disability was scored using the pediatric stroke outcome measure (PSOM 0 [no deficit] to 10 [maximum deficit]).^18^ For patients with missing PSOM values, the coordinating investigator (M.K.) established a retrospective scoring inferred from medical records in collaboration with the local investigators.^11,12^

### Statistical Analysis

Continuous variables were summarized using means (SDs) or medians (IQRs) as appropriate, and discrete variables were summarized using counts (percentages). Variables were summarized descriptively by treatment type for all patients included, and no imputation of missing variables was performed. We used the χ^2^ or Fisher exact test, as appropriate, for comparisons of categorical variables between treatment groups and among age groups (0-6, 7-12, and 13-18 years) and we used the Wilcoxon rank sum test for continuous variables. All computations were performed with JMP, version 14 (SAS Institute Inc). The significance threshold was set at a 2-tailed value of *P* < .05 for all analyses. If needed, we derived 95% CIs by bootstrapping (2500 occurrences) statistical results.^19^

## Results

### Characteristics at the Acute Phase

Baseline characteristics of the population, including missing data, are presented in Table 1. Overall, 68 children from 29 participating centers were analyzed, including 24 girls (35.3%) and 44 boys (64.7%), with a median age at stroke onset of 11 (IQR, 4-16) years and body weight of 42 (IQR, 20-60) kg. History of varicella was found in 12.5% (6 of 48 patients with available data) and potentially embolic heart condition in 16.2% (11). Median pedNIHSS at admission was 13 (IQR, 7-19) and was higher in patients treated with EVT (16 [IQR, 10-20] vs 9 [IQR, 6-17]; *P* = .004). Median time from stroke onset to imaging was 2 hours and 54 minutes (IQR, 2 hours to 4 hours and 49 minutes) and was longer in patients treated with EVT (3 hours and 7 minutes; IQR, 2 hours and 3 minutes to 6 hours and 24 minutes vs 2 hours and 39 minutes; IQR, 1 hour and 51 minutes to 4 hours and 13 minutes; *P* = .04). Admission imaging modality was MRI in 53 patients (77.9%) and CT in 15 patients (22.1%).

**Table 1.** Baseline Characteristics and Outcome

Variables	Patients, No./total No. (%)			*P* value
	Total (n = 68)	IVT only (n = 28)	EVT (n = 40)	
**Patient characteristic**				
Age at stroke onset, median (IQR), y	11 (4-16)	12 (5-16)	11 (4-16)	.64
Sex				.80
Female	24 (35.3)	9 (32.1)	15 (37.5)	
Male	44 (64.7)			
Hypertension, diabetes	0	0	0	NA
Tobacco use (current or past)	4/39 (10.3)	0/18	4/21 (19.0)	.05
History of thrombophilia	2/63 (3.2)	1/25 (4.2)	1/38 (2.6)	.55
History of genetic disorder	4/66 (6.1)	1 (3.6)	3/38 (7.9)	.47
History of varicella <1 y	6/48 (12.5)	2/20 (10.0)	4/28 (14.3)	.66
History of infection <6 mo	9 (13.2)	4 (14.3)	5 (12.5)	.83
Current infection	9 (13.2)	3 (10.7)	6 (15.0)	.61
History of stroke or TIA	9 (13.2)	3 (10.7)	6 (15.0)	.76
History of embolic heart disease	11 (16.2)	5 (17.9)	6 (15.0)	.75
Pre-stroke mRS ≥1	7 (10.3)	2 (7.1)	5 (12.5)	.47
**Stroke characteristics and management**				
Unwitnessed onset or wake-up stroke	4 (5.9)	1 (3.6)	3 (7.5)	.50
Transfer before treatment	17 (25.0)	0	17 (42.5)	<.001
Baseline pedNIHSS, median (IQR), No.	13 (7-19)	9 (6-17)	16 (10-20)	.004
Baseline ASPECTS, median (IQR), No.	8 (6-9) (n = 66)	8 (7-9) (n = 26)	7 (5-9) (n = 40)	.24
Baseline core volume (ABC/2), median (IQR), No., mL	10.3 (2.3-32.8) (n = 64)	9.1 (2-19.8) (n = 27)	12 (3.3-49.3) (n = 37)	.53
IVT administration	44 (64.7)	28 (100)	16 (40.0)	<.001
**Occlusion location**				
Anterior circulation	57 (83.8)	24 (85.7)	33 (82.5)	.43
Internal carotid artery	17/57 (29.8)	5 (17.9)	12 (30)	.09
Middle cerebral artery, M1 segment	29/57 (50.9)	11 (39.3)	18 (45)	
Other anterior location	11/57 (19.3)	8 (28.6)	3 (7.5)	
Posterior circulation	11 (16.2)	4 (14.2)	7 (17.5)	.86
Basilar artery	6/11 (54.5)	0	6 (15)	.17
Posterior cerebral artery, P1 segment	2/11 (18.2)	2 (7.1)	0	
Other posterior location	3/11 (27.3)	3 (10.7)	0 (0)	
Tandem occlusion	6/53 (11.3)	1/19 (5.3)	5/34 (14.7)	.40
**Time metrics**				
Symptom onset to imaging, median (IQR), No., h^a^	2 h, 54 min (2 h-4 h, 49 min) (n = 67)	2 h, 39 min (1 h, 51 min- 4 h, 13 min) (n = 28)	3 h, 7 min (2 h, 03 min-6 h, 24 min) (n = 39)	.04
Symptom-onset to IVT, median (IQR), h^a^	3 h, 30 min (2 h, 33 min-4 h, 8 min) (n = 41)	3:44 (2:41-4:28) (n = 26)	3 h, 15 min (2 h-3 h, 55 min) (n = 15)	.15
Symptom-onset to groin puncture, median (IQR), No., h^a^	NA	NA	5 h, 28 min (4 h, 26 min-8 h, 13 min) (n = 36)	NA
Symptom-onset to final mTICI, median (IQR), No., h^a^	NA	NA	6 h, 25 min (5 h, 38 min-8 h, 41 min) (n = 36)	NA
**Stroke etiology (CASCADE)**				
Cardioembolic	21 (30.9)	7 (25.0)	14 (35.0)	.56
Focal cerebral arteriopathy	17 (25.0)	9 (32.1)	8 (20.0)	
Aortic/cervical arteriopathy	9 (13.2)	0	9 (22.5)	
Thrombotic	8 (11.8)	4 (14.3)	4 (10.0)	
Other intracranial arteriopathy	3 (4.4)	2 (7.1)	1 (2.5)	
Other etiology	2 (2.9)	1 (3.6)	1 (2.5)	
Undetermined etiology	8 (11.8)	5 (17.9)	3 (7.5)	
**Outcome measures**				
Postprocedural successful reperfusion (mTICI 2b-3)	NA	NA	32 (80)	NA
24 h, ASPECTS, median (IQR)	7 (4-8) (n = 55)	8 (6-9) (n = 21)	5 (3-7) (n = 34)	.01
24 h, core volume (ABC/2), median (IQR), No., mL	14.5 (5.8-60.6) (n = 54)	11.5 (5-23.1) (n = 22)	37.5 (6.6-75) (n = 32)	.11
24 h, delta core volume (ABC/2), median (IQR), No., mL	1.4 (0-9.7) (n = 50)	0.9 (0-4.5) (n = 21	2.7 (0-29.9) (n = 29	.29
24 h, pedNIHSS, median (IQR), No.	5 (2-15) (n = 67)	4 (1-14) (n = 28)	9 (3-16) (n = 39)	.12
24 h, pedNIHSS reduction, median (IQR), No.	4 (0-9) (n = 67)	3 (1-6) (n = 28)	6 (0-9) (n = 39)	.28
Day-7, pedNIHSS, median (IQR), No.	3 (1-8) (n = 65	1 (0-5) (n = 26)	5 (2-11) (n = 39)	.13
Day7, pedNIHSS reduction, median (IQR), No.	7 (4-11) (n = 65)	6 (4-10) (n = 26)	7 (4-11)	.26
Day 90, PSOM, median (IQR), No.	2 (0.5-4) (n = 61)	0.5 (0-2) (n = 23)	1.8 (0.9-3.6) (n = 38)	.02
Day 90, mRs 0-1	33/67 (49.3)	16/27 (59.3)	17 (42.5)	.22
Day 90, mRS 0-2	46/67 (68.7)	21/27 (77.8)	25 (62.5)	.28
Day 90, mRS 0-3	60/67 (89.6)	24/27 (88.9)	36 (90.0)	>.99
Day 90, mortality	3/67 (4.5)	2/27 (7.4)	1 (2.5)	.35
1-y, PSOM, median (IQR), No.	1.5 (0.5-3) (n = 60)	0.5 (0-2) (n = 23)	1.5 (0.5-3) (n = 37)	.03
1 y, mRS 0-1	35/66 (53)	18/27 (66.7)	17/39 (43.6)	.08
1 y, mRS 0-2	52/66 (78.8)	21/27 (77.8)	31/39 (79.5)	>.99
1 y, mRS 0-3	58/66 (87.9)	23/27 (85.2)	35/39 (89.7)	.58
1 y, mortality	3/66 (4.5)	2/27 (7.4)	1/39 (2.6)	.35
ICH	4/56 (7.1)	0/22	4/34 (11.8)	.15
sICH	1/56 (1.8)	0/22	1/34 (2.9)	.84
Craniectomy	4/33 (12.1)	1/12 (8.3)	3/21 (14.3)	.56

Abbreviations: ASPECTS, Alberta Stroke Program Early Computed Tomography Score; CASCADE, Childhood Arterial Ischemic Stroke Standardized Classification and Diagnostic Evaluation; EVT, endovascular treatment; ICH, intracerebral hemorrhage; IVT, intravenous thrombolysis; M1, proximal segment of the middle cerebral artery; mRS, modified Rankin Scale; mTICI, modified treatment in cerebral infarction; NA, not applicable; pedNIHSS, Pediatric National Institutes of Health Stroke Scale; P1, proximal segment of the posterior cerebral artery; PSOM, pediatric stroke outcome measure; sICH, symptomatic intracerebral hemorrhage (ECASS II); TIA, transient ischemic attack.

^a^ Or last-seen well.

Acute ischemic stroke involved the anterior circulation in 57 children (83.8%) and 11 (16.2%) had a posterior circulation stroke. Proximal occlusion was encountered in 52 (76.5%) of patients overall, (16 of 28 patients (57.1%) of received IVT alone and 37 of 40 (92.5%) received EVT). Table 1 provide further details. Median baseline ASPECTS score was 8 (IQR, 6-9) and core volume (ABC/2 method) was 10.3 (IQR, 2.3-32.8) mL; both variables did not differ significantly between patients treated with or without EVT.

Regarding the final diagnosis of stroke etiology, 17 patients (25.0%) had an FCA, 9 patients (13.2%) had aortic/cervical arteriopathy (arterial dissection in all cases), and 21 patients (30.9%) had a cardioembolic cause. The distribution of etiology was not significantly different between patients treated with and without EVT.

### Revascularization Treatments

Three procedures were performed at children’s hospitals and 37 were conducted in adult hospitals. All physicians performing the procedures were senior interventional neuroradiologists with at least 3 years of experience in EVT working in tertiary adult centers.

Intravenous thrombolysis was administered in 44 of 68 patients (64.7%), alone (28 [41.2%]) or before EVT (16 [23.5%]). Endovascular treatment was performed in 40 children (58.8%). Median time from onset to IVT was 3 hours and 30 minutes (IQR, 2 hours and 33 minutes to 4 hours and 28 minutes) and did not differ significantly between treatment groups (3 hours and 44 minutes; IQR, 2 hours and 41 minutes to 4 hours and 28 minutes) with IVT vs 3 hours and 15 minutes (IQR, 2 hours to 3 hours and 55 minutes) with EVT; *P* = .15).

Most of the EVT procedures were performed under general anesthesia (33 of 39 [84.6%]), with a median onset to groin puncture of 5 hours and 28 minutes (IQR, 4 hours and 26 minutes to 8 hours and 13 minutes), and onset to reperfusion of 6 hours and 25 minutes (IQR, 5 hours and 38 minutes to 8 hours and 41 minutes). Revascularization therapy and stroke diagnostic procedures occurred in the same hospital for 53 of 70 procedures (75.7%).

Spontaneous or postintravenous r-tPA reperfusion greater than or equal to mTICI 2b was seen at the beginning of the EVT procedure in 4 of 40 (10.0%) patients with no additional endovascular treatment. Mechanical thrombectomy with a stent retriever was the most common first-line approach in 19 of 34 patients (55.9%), followed by contact aspiration in 11 of 34 patients (32.4%), and the combined technique in 4 of 34 (11.8%). Switching to another endovascular approach (rescue therapy) occurred in 6 patients, including balloon angioplasty in 1 child. Complementary intraarterial thrombolysis was used in 2 children.

## Outcomes

### Recanalization

**Figure 1.**
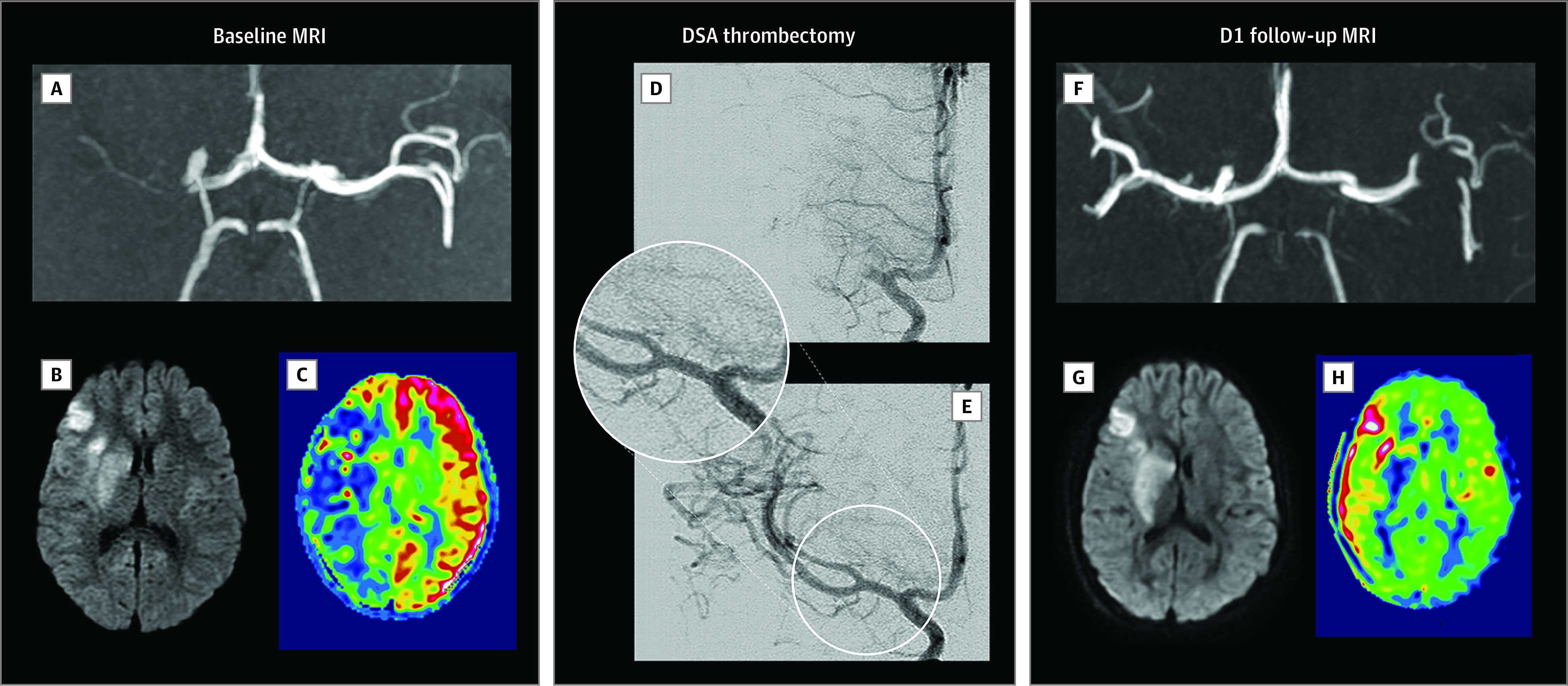
Pediatric Acute Ischemic Stroke of Cardioembolic Origin Treated With Mechanical Thrombectomy Acute ischemic stroke in a child aged 8 years with a clinical history of embolic heart disease presenting with sudden left hemiplegia and dysarthria. A-C: Baseline magnetic resonance imaging (MRI) performed 5 hours and 20 minutes after stroke onset. A, Axial maximum intensity projection reconstruction of time-of-flight angiography showing the occlusion of the proximal M1 segment of the right middle cerebral artery (MCA). B, Axial diffusion image showing hyperintensity limited to right caudate and lenticular nuclei, insula, and frontal cortex. C, Axial arterial-spin-labeling with cerebral blood flow cartography (ASL-CBF) showing markedly decreased perfusion throughout the right sylvian territory compared with the left side. D-E: Periprocedural digital subtraction angiography (DSA) images. D, Initial anteroposterior images after opacification of the right carotid territory confirming the occlusion (proximal M1 segment) of the right MCA. E, Final anteroposterior images after mechanical thrombectomy with stent retriever showing the patency without stenosis of the right MCA. F-H: Follow-up MRI performed 24 hours (D1) after the mechanical thrombectomy. F, Axial maximum intensity projection reconstruction of time-of-flight angiography showing restored patency of the right MCA territory. G, Axial diffusion image with no major extension of the ischemic core. H, Axial ASL-CBF cartography showing restored perfusion in the right MCA territory.

**Figure 2.**
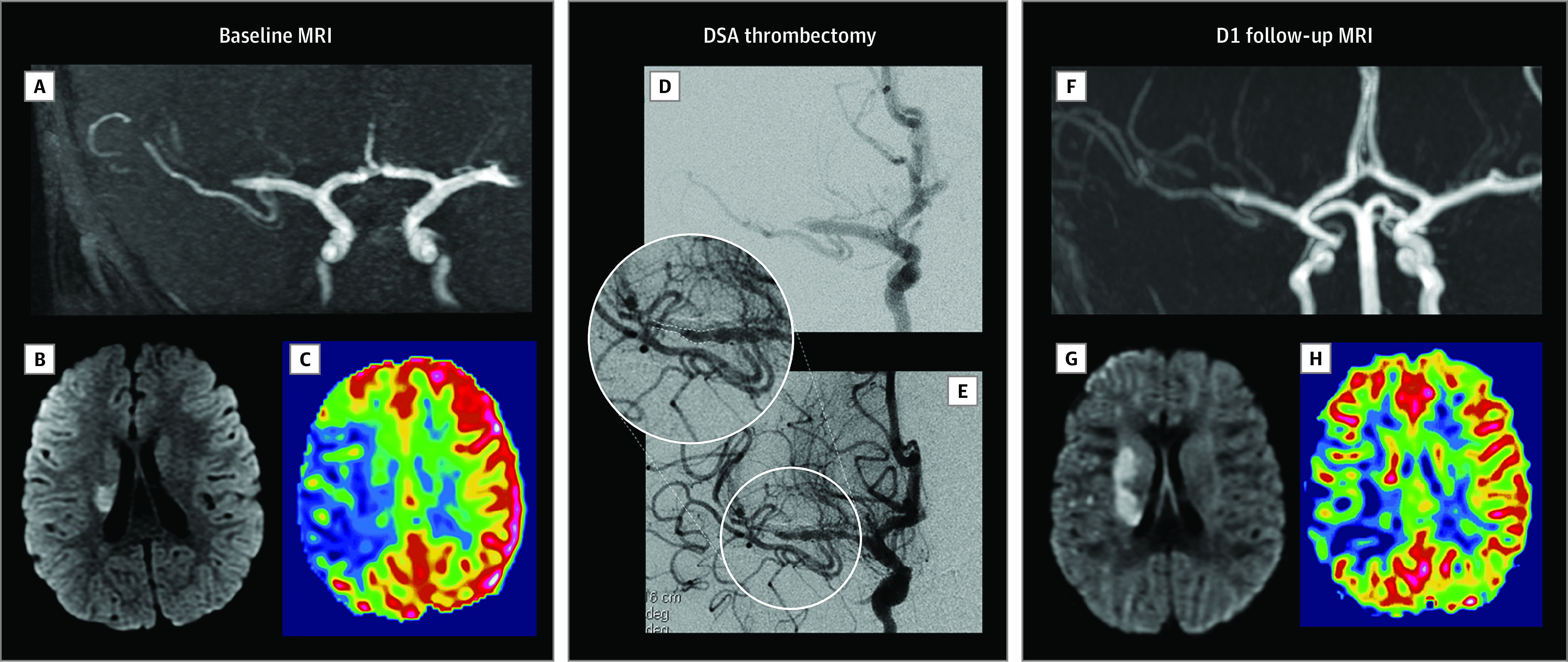
Pediatric Acute Ischemic Stroke Caused by FCA Treated With Mechanical Thrombectomy Acute ischemic stroke in a child aged 4 years with no medical history, presenting as a sudden left hemiplegia and facial palsy. A-C: Baseline magnetic resonance imaging (MRI) performed 2 hours and 50 minutes after stroke onset. A, Coronal maximum intensity projection reconstruction of time-of-flight angiography showing the occlusion of the distal M1 segment of the right middle cerebral artery (MCA). B, Axial diffusion image showing hyperintensity limited to right caudate. C, axial arterial spin labeling with cerebral blood flow (ASL-CBF) cartography showing markedly decreased perfusion throughout the right sylvian territory compared with the left side. D-E: Periprocedural digital subtraction angiography (DSA) images. D, Initial anteroposterior images after opacification of the right carotid territory confirming the occlusion (distal M1 segment) of the right MCA. E, Final anteroposterior images after mechanical thrombectomy with stent retriever showing restored patency of the right MCA; however, with significant residual stenosis. F-H: Follow-up MRI performed 24 hours (D1) after the mechanical thrombectomy. F, Axial MIP reconstruction of time-of-flight images showing the reclusion of the stenosed MCA. G, Axial diffusion image showing the extension of the ischemic core to the caudate nucleus and the posterior limb of the internal capsule; note the small focal ischemic spots in the superficial MCA territory. H, Axial ASL-CBF cartography showing the remaining decreased perfusion in the right MCA territory.

In patients undergoing EVT, postprocedural angiographic outcome was good (≥mTICI 2b) in 80% of the procedures (32 of 40: 13 mTICI3, 2 mTICI2c, 17 mTICI 2b) and in 77.8% (28 of 36) of mechanical thrombectomy performed. A first-pass recanalization was noted in 12 of 35 patients (34.3%) with a median of 2 passes (IQR, 1-3). Although the rate of successful postprocedural angiographic recanalization (mTICI 2b-3) did not differ substantially among the main stroke causes (Table 1), persistent proximal arterial stenosis greater than or equal to 50% on final digital subtraction arteriography images was significantly more frequent in FCA compared with cardioembolic and dissection procedures (3 of 6 [50.0%] vs 0 of 23; *P* = .009). Likewise, in follow-up MRA or CTA 24 hours after mechanical thrombectomy, arterial reocclusion or stenosis greater than or equal to 50% was more frequent in patients with FCA (5 of 7 [71.4%]) vs cardioembolic and dissection-related stroke (3 of 13 [23.1%]) (*P* = .04) (Figure 1 and Figure 2).

In the EVT group, 1 patient developed intracranial arterial perforation (2.7%; 95% CI, 0%-16.6%), and 1 developed emboli in a new territory; both of these children had unfavorable outcomes at 1 year (mRs, 4). No other vascular complications, such as arterial dissection, periprocedural thrombosis, or puncture site complications were reported.

### Early Clinical and Radiologic Outcomes

Death was observed in 3 patients (4.4%; 95% CI, 0%-12.1%), all within the first week (2 malignant infarctions and 1 major symptomatic intracerebral hemorrhage). Median pedNIHSS reduction at 24 hours was 4 (IQR, 0-9) points, and was slightly but not significantly better in the EVT group (6; IQR, 0-9 vs 3; IQR, 1-6 points; *P* = .28). Intracerebral hemorrhage was observed in 4 patients (7.1%; 95% CI, 3.5%-16%) and symptomatic intracerebral hemorrhage was observed in 1 patient (1.8%; 95% CI, 0%-8.9%), all in the EVT group. Four malignant infarctions had decompressive hemicraniectomy.

### Long-term Clinical Outcome

Long-term clinical outcome was good, with an mRS score of 0 to 2 noted in 46 of 67 children (68.7%) at 3 months and 52 of 66 (78.8%) at 12 months after stroke onset. The median mRS score was 2 (IQR, 0-3) at 3 months and 1 (IQR, 0-2) at 12 months and did not differ significantly between the EVT and IVT-only groups. The median PSOM score was 2 (IQR, 0.5-4) after 3 months and 1.5 (IQR, 0.5-3) after 12 months—not significantly different between the EVT and IVT-only groups. Analysis by age group suggests that stroke recovery may be longer in children younger than 6 years without an association with longer-term prognosis with higher mRS scores in this subgroup at 3 months but comparable to scores in older children at 12 months (Table 2).

**Table 2.** Baseline Characteristics and Outcome by Age Group

Variables	Patients, No./total No. (%)^a^				*P* value
	Total (n = 68)	0-6 y (n = 23)	7-12 y (n = 13)	13-18 y (n = 32)	
**Patient characteristic**					
Age at stroke onset, median (IQR), No., y	11 (4-16)	2 (2-5)	10 (8-11)	16 (14-16)	
Sex					
Female	24 (35.3)	10 (43.5)	3 (23.1)	11 (34.4)	.46
Male	44 (64.7)	13 (56.5)	10 (76.9)	21 (65.6)	
Hypertension, diabetes	0	0	0	0	
Tobacco use (current or past)	4/39 (10.3)	0/2	0/8	4/29 (13.8)	.46
History of thrombophilia	2/63 (3.2)	1/22 (4.5)	0/13	1/28 (3.6)	.75
History of genetic disorder	4/66 (6.1)	1/22 (4.5)	1/12 (8.3)	2/32 (6.3)	.91
History of varicella <1 y	6/48 (12.5)	6/16 (37.5)	0/11	0/21	.001
History of infection <6 mo	9 (13.2)	7 (30.4)	0	2 (6.3)	.009
Current infection	9 (13.2)	7 (30.4)	1 (7.7)	1 (3.1)	.01
History of stroke or TIA	9 (13.2)	5 (21.7)	2 (15.4)	2 (6.3)	.13
History of embolic heart disease	11 (16.2)	6 (26.1)	1 (7.7)	4 (12.5)	.26
Prestroke mRS ≥1	7 (10.3)	4 (17.4)	2 (15.4)	1 (3.1)	.18
**Stroke characteristics and management**					
Unwitnessed onset or wake-up stroke	4 (5.9)	2 (8.6)	1 (7.7)	1 (3.2)	.02
Transfer before treatment	17 (25.0)	6 (26.1)	3 (23.1)	8 (25)	.98
Baseline pedNIHSS, median (IQR), No.	13 (7-19)	15 (9-19) (n = 23)	11 (7-20) (n = 13)	12 (7-17) (n = 32)	.73
Baseline ASPECTS, median (IQR), No.	8 (6-9) (n = 66)	7 (2-9) (n = 23)	7 (3-9) (n = 13)	8 (4-9) (n = 30)	.49
Baseline core volume (ABC/2), median (IQR), No., ml	10.3 (2.3-32.8) (n = 64)	16.7 (1.4-61.1) (n = 21)	16 (4.8-29) (n = 13)	9.1 (2-20.8) (n = 30)	.54
IVT administration	44 (64.7)	11 (47.8)	10 (76.9)	23 (71.9)	.11
Mechanical thrombectomy	40 (58.8)	15 (65.2)	7 (53.8)	18 (56.3)	.74
**Time metrics**					
Symptom-onset to imaging, median (IQR), No., h^b^	2 h, 54 min (2 h-4 h, 49 min) (n = 67)	4 h, 24 min (2 h, 32 min-6 h, 56 min) (n = 23)	2 h, 42 min (2 h, 8 min-5 h, 55 min) (n = 12)	2 h, 38 min (1 h, 43 min-3 h, 52 min) (n = 32)	.02
Symptom-onset to IVT, median (IQR), h^b^	3 h, 30 min (2 h, 33 min-4 h, 8 min) (n = 41)	3 h, 45 min (3 h, 35 min-4 h, 27 min) (n = 11)	4 h, 00 (3 h, 09-4 h, 36) (n = 10)	2 h, 50 min (1 h, 51 min-3 h, 36 min) (n = 20)	.02
Symptom-onset to groin puncture, median (IQR), No., h^b^	5 h, 28 (4 h, 26 min-8 h, 13 min) (n = 36)	8 h, 24 (5 h, 30 min-14 h, 29 min) (n = 14)	5 h, 05 (4 h, 15-5 h, 30) (n = 7)	4 h, 58 min (4 h, 22 min −5 h, 26 min) (n = 15)	.02
Symptom-onset to final mTICI, median (IQR), No., h^b^	6 h, 25 min (3 h, 38 min-8 h, 41 min) (n = 36)	8 h, 56 (6 h, 27-15 h, 10) (n = 13)	5:47 (4:35-6:25) (n = 7)	6 h, 4 min (5 h, 35 min-6 h, 36 min) (n = 16)	.02
**Stroke etiology (CASCADE)**					
Cardioembolic	21 (30.9)	8 (34.8)	2 (15.4)	11 (34.4)	.38
Focal cerebral arteriopathy	17 (25.0)	7 (30.4)	4 (30.8)	6 (18.8)	
Aortic/cervical arteriopathy	9 (13.2)	4 (17.4)	2 (15.4)	3 (9.4)	
Thrombotic	8 (11.8)	1 (4.3)	2 (15.4)	5 (15.6)	
Other intracranial arteriopathy	3 (4.4)	0	2 (15.4)	1 (3.1)	
Other etiology	2 (2.9)	0	1(7.7)	1 (3.1)	
Undetermined etiology	8 (11.8)	3 (13)	0	5 (15.6)	
**Outcome measures**					
Postprocedural successful reperfusion (mTICI 2b-3)	32/40 (80.0)	11/15 (73.3)	7/7 (100)	14/18 (77.8)	.35
24 h, ASPECTS, median (IQR)	7 (4-8) (n = 55)	5 (2-8) (n = 19)	7 (3-9) (n = 12)	7 (1-9) (n = 24)	.09
24 h, pedNIHSS, median (IQR), No.	5 (2-15) (n = 67)	12 (5-17) (n = 23)	4 (2-16) (n = 13)	3 (1-11) (n = 31)	.02
24 h, pedNIHSS reduction, median (IQR), No.	4 (0-9) (n = 67)	2 (0-6) (n = 23)	3 (2-8) (n = 13)	6 (2-10) (n = 31)	.07
Day 7, pedNIHSS, median (IQR), No.	3 (1-8) (n = 65)	7 (2-12) (n = 21)	4 (1-8) (n = 13)	2 (0-6) (n = 31)	.05
Day 7, pedNIHSS reduction, median (IQR), No.	7 (4-11) (n = 65)	5 (1-10) (n = 21)	8 (5-13) (n = 13)	8 (5-13) (n = 31)	.28
Day 90, PSOM, median (IQR), No.	2 (0.5-4) (n = 61)	1.5 (0.9-4.3) (n = 21)	1 (0-2.5) (n = 11)	1 (0.5-2.5) (n = 29)	.29
Day 90, mRs 0-1	33/67 (49.3)	10 (43.5)	6 (46.2)	17/31 (54.8)	.69
Day 90, mRS 0-2	46/67 (68.7)	13 (56.5)	11 (84.6)	22/31 (71)	.21
Day 90, mRS 0-3	60/67 (89.6)	17 (73.9)	13 (100)	30/31 (96.8)	.009
Day 90, mortality	3/67 (4.5)	2 (8.7)	0	1/31 (3.2)	.43
1 y, PSOM, median (IQR), No.	1.5 (0.5-3) (n = 60)	1.5 (0.5-3) (n = 21)	1.5 (0.1-3.3) (n = 12)	0.8 (0.4-2.1) (n = 27)	.55
1 y, mRS 0-1	35/66 (53)	11 (47.8)	7 (53.8)	17/30 (56.7)	.81
1 y, mRS 0-2	52/66 (78.8)	17 (73.9)	11 (84.6)	24/30 (80)	.73
1 y Mortality	3/66 (4.5)	2 (8.7)	0	1/30 (3.3)	.44
ICH	4/56 (7.1)	3/19 (15.8)	1/12 (8.3)	0/25	.13
sICH	1/56 (1.8)	1/19 (5.3)	0/12	0/25	.37
Craniectomy	4/33 (12.1)	0/13	1/4 (25.0)	3/16 (18.8)	.21

Abbreviations: ASPECTS, Alberta Stroke Program Early Computed Tomography Score; CASCADE, Childhood Arterial Ischemic Stroke Standardized Classification and Diagnostic Evaluation; ICH, intracerebral hemorrhage; IVT, intravenous thrombolysis; mRS, modified Rankin Scale; mTICI, modified Treatment in Cerebral Infarction; pedNIHSS, Pediatric National Institutes of Health Stroke Scale; PSOM, pediatric stroke outcome measure; sICH, symptomatic intracerebral hemorrhage (ECASS II); TIA, transient ischemic attack.

^a^ Total No. in No./total No. (%) differs from column heading when varying amounts of data were available.

^b^ Or last-seen well.

### Recruiting Centers and Method Validation

In mainland France and overseas territories, 57 academic hospitals were contacted through their respective stroke center, pediatric neurology, and interventional radiology (where applicable) departments. The response rate was 100%. Twenty-eight centers (49.1%) reported no revascularization treatment in pediatric patients with AIS during the study period, and 29 centers reported at least 1 treatment. A median of 2 patients (IQR, 1-3), with a maximum of 8, were treated at each center during the 42-month inclusion period.

In the validation region, 21 centers were contacted (9 academic and 12 nonacademic centers). Among the 17 patients included in this region, only 1 was treated in a nonacademic center, yielding a transposed estimated 94% (95% CI, 73.0%-98.9%) nationwide exhaustivity during the study period.

## Discussion

This study provides data on acute revascularization strategies for AIS in select children, using a retrospective nationwide study initiated in 2015, when mechanical thrombectomy became the standard of care in adults with AIS with large-vessel occlusion, alone or in addition to IVT. Our main findings are that (1) complication rates were low in a population of children with AIS treated with IVT, EVT, or both, similar to rates seen in the largest clinical prospective adult registries^20^; (2) EVT was feasible with rates of successful revascularization of similar magnitude to those found in adult populations^21^; (3) patients with cardioembolic stroke were overrepresented and had overall better angiographic results; (4) 1-year neurologic outcome was good in three-quarters of the study sample; and (5) the long-term outcome was not substantially different between the EVT and no-EVT groups in this cohort, despite higher initial severity markers in the mechanical thrombectomy group. These results provide data on the safety of acute revascularization strategies in children and provide additional key insight on the role and indications of these therapeutic approaches in pediatric age groups.

Although recanalization treatments, including intravenous fibrinolysis and endovascular thrombectomy are the standard of care for select adults with AIS- large-vessel occlusion,^3,17^ high-level evidence is lacking in pediatric AIS large-vessel occlusion, and data remain scarce. Resolving this issue is likely a long-term effort. In line with the much lower incidence of AIS in children than in adults, decreased awareness of treatment options among nonstroke-oriented pediatric neurologists, limited availability of acute pediatric EVT in most pediatric centers, and increased delays owing to the difficulty to identify early clinical symptoms of pediatric stroke, especially in younger children, limit the implementation of revascularization treatments. Moreover, there are important issues of concern among the pediatric community concerning revascularization treatment safety in children, linked with pediatric specificities in stroke etiologies, maturation of the coagulation system, experience of clinicians, and size of the devices for small children EVT. Presumably, these factors also largely contributed to recruitment failure of the Thrombolysis in Pediatric Stroke (TIPS) Study,^4^ leading to its discontinuation and questioning the future steps for improvement for pediatric AIS. In the TIPS study, the authors pointed out that only 1 patient was enrolled for every 93 patients screened, and that after excluding half of alternative nonischemic diagnoses, most exclusions were based on contraindications for use of r-tPA, potentially sending an indirect indication relating to a perceived complexity of the use of r-tPA in children.^4^ More recently though, the extended results of the TIPS trial were published, providing additional data on children treated at TIPS sites, and reporting the outcomes of 26 children treated between 2013 and 2018.^22^ In this work, the authors noted that no child experienced a symptomatic hemorrhagic transformation following r-tPA infusion and that asymptomatic intracerebral hemorrhage occurred very infrequently compared with the incidence rates in adult samples. Similarly, in our sample, none of the children treated with PA developed intracranial hemorrhage or neurologic deterioration following r-tPA administration. Together, our data are in line with previous reports that the risk of ICH after intravenous r-tPA is low in in appropriately selected children,^4,22^ suggesting that for similarly selected children, the benefits of thrombolysis may outweigh the risks.

Moving forward, as discussed in the recently published study on the Save ChildS registry,^9^ the main drawback of the off-label use of EVT in children is concern regarding its safety in the absence of solid evidence regarding the clinical benefits of EVT.^5^ While data concerning the safety profile of r-tPA in the pediatric population are notable, safety of pediatric EVT remains debated. The continuous improvement of devices and techniques, issued from adult experience and of pediatric stroke pathway of care may limit comparability of groups over time.^5^

In this study, 4 of 34 children (11.8%) treated with EVT demonstrated hemorrhagic transformation, and only 1 of 40 children (2.5%) experienced neurologic deterioration. This figure is of similar magnitude to that found in the SaveChildS registry,^9^ where 1 of 73 patients (1.3%) developed a symptomatic intracranial hemorrhage following EVT. Less-recent reports by Bigi et al^8^ and Satti et al^23^ also described or aggregated safety outcomes in similar ranges, but included patients treated at times in which EVT was not standard of care, even in the adult population, with notably different strategies for endovascular approaches, such as children treated only with intraarterial infusion of r-tPA, precluding relevant comparisons. Overall, symptomatic intracerebral hemorrhage occurred in 1 patient (1.8%) in the KidClot study vs 4.4% patients in the HERMES adult meta-analysis,^3^ but this result should be interpreted with caution because the 95% CI is quite large (0%-8.9%) in the KidClot study because of the limited population size.

Further substantiating the apparent safety of revascularization approaches, among the 40 children treated with EVT in this cohort, there was only 1 occurrence of vessel perforation (2.7%). The perforation happened in a 2-year-old child with a cardioembolic stroke and occlusion of the distal M1 segment of the middle cerebral artery. Mechanical thrombectomy was performed with a stent retriever (size 4 × 20 mm), which is quite a large stent compared with the size of the vessels in children this age, but it was the smallest size available in the center in 2013 (date of the procedure). The blood leak was closed by glue injection, but unfortunately the clinical outcome was not good (mRS, 4 at 12 months). For comparison, vessel perforation was reported in 1.7% of patients in a large adult EVT registry^20,24^; however, caution is warranted given the 95% CI range for this complication in the KidClot study.

One important safety question is whether certain underlying etiologies bear a higher risk for EVT procedure complications or futility. In our study, as in the SaveChildS study,^9^ the representation of stroke etiologies is different from classically reported causes of stroke, with fewer children with focal cerebral arteriopathy (25% in KidClot, 10% in SaveChilds, 30% in overall pediatric strokes) and more children with a cardioembolic stroke.^5^ This variability may reflect the widely expressed concern about EVT in the setting of FCA, relating to the inflammatory nature of FCA, which may represent a risk factor for artery perforation or treatment futility. In our study, patients with FCA displayed a low procedural hemorrhagic risk comparable to patients with cardioembolic stroke. the rate of reocclusion or persisting more than 50% stenosis on follow-up imaging was substantially elevated in this subgroup. These results suggest a higher possibility of EVT treatment failure, but with safety, in patients with FCA, which should be further investigated.

Furthermore, included patients were older than overall pediatric patients with AIS in both reports, potentially suggesting that (1) treating physicians were likely more inclined to use IVT or EVT in older children, who resemble adults, (2) treating physicians made an implicit choice toward patients with cardioembolic stroke (who are older than those with FCA), and (3) younger children may have been deemed either ineligible or presented outside the standard therapeutic windows owing to increased care delays.

Although our data contribute to the accumulating evidence on the role of emergent revascularization treatments for children with acute cerebral arterial occlusion, there remain issues that will likely require long-term international collaboration on the way to optimizing therapeutic strategies in pediatric patients. One key issue regards the benefit of emergent revascularization therapies in this population.

If the proportion of children with AIS who could theoretically benefit from revascularization is largely unknown, we believe the children in our cohort who were treated during the study period represent the overall population among a pool of 15 million individuals younger than 18 years.^25^ Considering an incidence rate of 2 to 8 per 100 000 children-years for all pediatric AIS,^1^ this rate translates to a proportion of 1.5% to 5.9% of children with AIS receiving revascularization. This figure compares unfavorably with the rates of more than 40% of eligibility for revascularization found in adults, following the initiation of both telestroke networks and expansion in EVT indications.^26^ It is arguable that in the absence of robust evidence for the efficacy of these newer strategies, this proportion will not steadily increase, but our study aimed to provide data for both clinicians admitting children with suspected AIS and policy makers to inform future treatment recommendations. Yet, as moving forward, the community needs to address the question as to why revascularization strategies are proposed at such low rates in children, limiting both the opportunities to optimize systems of care locally^7^ and the feasibility of a randomized evaluation of treatment strategies.^4^ In this work, we found that 75% of included centers (encompassing all the largest volume stroke centers nationally) treated fewer than a median of 1 pediatric patient per year with IVT, EVT, or both strategies. This point mandates a careful analysis of potential means for improvement both locally and globally that could include standardized imaging pathways for children with acute neurologic symptoms and increase awareness at adult stroke centers and nontertiary pediatric emergency departments.

### Strengths and Limitations

The strengths of our study include (1) a relatively large sample size of recanalization treatment in pediatric patients, observed after mechanical thrombectomy became standard of care in adults with AIS and large-vessel occlusion, using modern devices and, in some cases, an optimized pathway; (2) a centralized review of imaging data; and (3) a centralized review of stroke etiology by a multidisciplinary symposium. The study has limitations. These limitations are inherent to the retrospective design, including missing data, selection bias, heterogeneity of the population, lack of a control group and, inability to perform direct comparisons between treatment groups.

## Conclusions

The findings of this study suggest that use of IVT, EVT, or both in a population of children with AIS may be safe. The 1-year neurologic outcome was good in three-quarters of the study sample. Endovascular treatment proved to be feasible, with rates of successful revascularization and procedural duration of similar magnitude to those found in adult populations. These results may help clinical decision-making for the use of revascularization treatment in pediatric AIS and encourage international collaboration to optimize therapeutic strategies and pathways in pediatric patients.

## Notes

Supplement. **Nonauthor Collaborators.** KidClot Group

## References

[1] Mallick AA, Ganesan V, Kirkham FJ, . Childhood arterial ischaemic stroke incidence, presenting features, and risk factors: a prospective population-based study. Lancet Neurol. 2014;13(1):35-43. doi:10.1016/S1474-4422(13)70290-4 24304598

[2] Lo W, Zamel K, Ponnappa K, . The cost of pediatric stroke care and rehabilitation. Stroke. 2008;39(1):161-165. doi:10.1161/STROKEAHA.107.497420 18032740

[3] Goyal M, Menon BK, van Zwam WH, ; HERMES collaborators. Endovascular thrombectomy after large-vessel ischaemic stroke: a meta-analysis of individual patient data from five randomised trials. Lancet. 2016;387(10029):1723-1731. doi:10.1016/S0140-6736(16)00163-X 26898852

[4] Rivkin MJ, deVeber G, Ichord RN, . Thrombolysis in pediatric stroke study. Stroke. 2015;46(3):880-885. doi:10.1161/STROKEAHA.114.008210 25613306 PMC4342311

[5] Chabrier S, Ozanne A, Naggara O, Boulouis G, Husson B, Kossorotoff M. Hyperacute recanalization strategies and childhood stroke in the evidence age. Stroke. 2021;52(1):381-384. doi:10.1161/STROKEAHA.120.031133 33349018

[6] Lansberg MG, Straka M, Kemp S, ; DEFUSE 2 study investigators. MRI profile and response to endovascular reperfusion after stroke (DEFUSE 2): a prospective cohort study. Lancet Neurol. 2012;11(10):860-867. doi:10.1016/S1474-4422(12)70203-X 22954705 PMC4074206

[7] Tabone L, Mediamolle N, Bellesme C, . Regional Pediatric Acute Stroke Protocol: Initial Experience During 3 Years and 13 Recanalization Treatments in Children. Stroke. 2017;48(8):2278-2281. doi:10.1161/STROKEAHA.117.016591 28546326

[8] Bigi S, Dulcey A, Gralla J, . Feasibility, safety, and outcome of recanalization treatment in childhood stroke. Ann Neurol. 2018;83(6):1125-1132. doi:10.1002/ana.25242 29679441

[9] Sporns PB, Sträter R, Minnerup J, . Feasibility, safety, and outcome of endovascular recanalization in childhood stroke: the Save ChildS Study. JAMA Neurol. 2020;77(1):25-34. doi:10.1001/jamaneurol.2019.3403 31609380 PMC6802048

[10] Dicpinigaitis AJ, Gandhi CD, Shah SP, . Endovascular thrombectomy with and without preceding intravenous thrombolysis for treatment of large vessel anterior circulation stroke: a cross-sectional analysis of 50,000 patients. J Neurol Sci. 2022;434:120168. doi:10.1016/j.jns.2022.120168 35101765

[11] Williams LS, Yilmaz EY, Lopez-Yunez AM. Retrospective assessment of initial stroke severity with the NIH Stroke Scale. Stroke. 2000;31(4):858-862. doi:10.1161/01.STR.31.4.858 10753988

[12] Kasner SE, Cucchiara BL, McGarvey ML, Luciano JM, Liebeskind DS, Chalela JA. Modified National Institutes of Health Stroke Scale can be estimated from medical records. Stroke. 2003;34(2):568-570. doi:10.1161/01.STR.0000052630.11159.25 12574577

[13] Barber PA, Demchuk AM, Zhang J, Buchan AM. Validity and reliability of a quantitative computed tomography score in predicting outcome of hyperacute stroke before thrombolytic therapy—ASPECTS Study Group; Alberta Stroke Programme Early CT Score. Lancet. 2000;355(9216):1670-1674. doi:10.1016/S0140-6736(00)02237-6 10905241

[14] Luby M, Hong J, Merino JG, . Stroke mismatch volume with the use of ABC/2 is equivalent to planimetric stroke mismatch volume. AJNR Am J Neuroradiol. 2013;34(10):1901-1907. doi:10.3174/ajnr.A3476 23449656 PMC4748711

[15] Bernard TJ, Manco-Johnson MJ, Lo W, . Towards a consensus-based classification of childhood arterial ischemic stroke. Stroke. 2012;43(2):371-377. doi:10.1161/STROKEAHA.111.624585 22156694 PMC3312781

[16] Goyal M, Fargen KM, Turk AS, . 2C or not 2C: defining an improved revascularization grading scale and the need for standardization of angiography outcomes in stroke trials. J Neurointerv Surg. 2014;6(2):83-86. doi:10.1136/neurintsurg-2013-010665 23390038 PMC4156591

[17] Hacke W, Kaste M, Fieschi C, ; Second European-Australasian Acute Stroke Study Investigators. Randomised double-blind placebo-controlled trial of thrombolytic therapy with intravenous alteplase in acute ischaemic stroke (ECASS II). Lancet. 1998;352(9136):1245-1251. doi:10.1016/S0140-6736(98)08020-9 9788453

[18] Kitchen L, Westmacott R, Friefeld S, . The pediatric stroke outcome measure: a validation and reliability study. Stroke. 2012;43(6):1602-1608. doi:10.1161/STROKEAHA.111.639583 22474056

[19] Efron B, Tibshirani RJ. An Introduction to the Bootstrap. CRC Press; 1994. doi:10.1201/9780429246593

[20] Happi Ngankou E, Gory B, Marnat G, ; ETIS Registry Investigators†. Thrombectomy complications in large vessel occlusions: incidence, predictors, and clinical impact in the ETIS Registry. Stroke. 2021;52(12):e764-e768. doi:10.1161/STROKEAHA.121.034865 34706564

[21] Goyal M, Saver JL, Ganesh A, . Standardized reporting of workflow metrics in acute ischemic stroke treatment: why and how? Stroke Vasc Interv Neurol. Published online November 17, 2021. doi:10.1161/SVIN.121.000177 PMC12512355 41584468

[22] Amlie-Lefond C, Shaw DWW, Cooper A, . Risk of intracranial hemorrhage following intravenous tPA (tissue-type plasminogen activator) for acute stroke is low in children. Stroke. 2020;51(2):542-548. doi:10.1161/STROKEAHA.119.027225 31842706

[23] Satti S, Chen J, Sivapatham T, Jayaraman M, Orbach D. Mechanical thrombectomy for pediatric acute ischemic stroke: review of the literature. J Neurointerv Surg. 2017;9(8):732-737. doi:10.1136/neurintsurg-2016-012320 27448827

[24] Madaelil TP, Kansagra AP, Cross DT, Moran CJ, Derdeyn CP. Mechanical thrombectomy in pediatric acute ischemic stroke: clinical outcomes and literature review. Interv Neuroradiol. 2016;22(4):426-431. doi:10.1177/1591019916637342 26945589 PMC4984382

[25] Institut de National De’Études Démographiques. Accessed August 15, 2022. https://www.ined.fr

[26] Vanacker P, Lambrou D, Eskandari A, Mosimann PJ, Maghraoui A, Michel P. Eligibility and predictors for acute revascularization procedures in a stroke center. Stroke. 2016;47(7):1844-1849. doi:10.1161/STROKEAHA.115.012577 27301945

