# Supplementary material for: Recanalization Treatments for Pediatric Acute Ischemic Stroke in France
Source: JAMA Netw Open. 2022 Sep 15;5(9):e2231343. doi: 10.1001/jamanetworkopen.2022.31343 (PMC9478769; doi:10.1001/jamanetworkopen.2022.31343)
Supplement: Supplement. — Nonauthor Collaborators. KidClot Group [file jamanetwopen-e2231343-s001.pdf]

Supplemental Online Content: Nonauthor Collaborators
*First name, last name, and suffix (if applicable) are required and will appear in PubMed.

*Group Name(s): KidClot Group							
***First Name and Middle Initial(s)**	***Last Name**	***Suffix (eg, Jr, III)**	Academic Degrees	Institution	Location (city, state/province, country)	Role or Contribution, eg, chair, principal investigator	Group (if more than 1 Group listed in the byline) and/or Subgroup (eg, Steering Committee)
WAGIH	BEN HASSEN		MD	w.benhassen@ghu-paris.fr	Paris / France	acquisition of Data	Kid Clot
MANOËLLE	KOSSOROTOFF		MD	manoelle.kossorotoff@aphp.fr	Paris / France	acquisition of Data	Kid Clot
OLIVIER	NAGGARA		MD	o.naggara@ghu-paris.fr	Paris / France	acquisition of Data	Kid Clot
BASILE	KERLEROUX		MD	b.kerleroux@ghu-paris.fr	Paris / France	acquisition of Data	Kid Clot
CHRISTIAN	DENIER		MD PhD	christian.denier@aphp.fr	Paris / France	acquisition of Data	Kid Clot
AUGUSTIN	OZANNE		MD	augustin.ozanne@aphp.fr	Paris / France	acquisition of Data	Kid Clot
CÉLINE	BELLESME		MD	celine.bellesme@aphp.fr	Paris / France	acquisition of Data	Kid Clot
BÉATRICE	HUSSON		MD	beatrice.husson@aphp.fr	Paris / France	acquisition of Data	Kid Clot
CHABRIAT	HUGUES		MD PhD	hugues.chabriat@aphp.fr	Paris / France	acquisition of Data	Kid Clot
REINER	PEGGY		MD	peggy.reiner@aphp.fr	Paris / France	acquisition of Data	Kid Clot
CATHERINE	LAMY		MD	c.lamy@ch-sainte-anne.fr	Paris / France	acquisition of Data	Kid Clot
FREDERIC	CLARENÇON		MD PhD	frederic.clarencon@aphp.fr	Paris / France	acquisition of Data	Kid Clot
SANDRINE	DELTOUR		MD PhD	sandrine.deltour@aphp.fr	Paris / France	acquisition of Data	Kid Clot
MICHÈLE	LEVASSEUR		MD	m.levasseur@ch-orsay.fr	Paris / France	acquisition of Data	Kid Clot
FRANÇOIS	LUN		MD	f.lun@gh-nord-essonne.fr	Paris / France	acquisition of Data	Kid Clot
HASSAN	HOSSEINI		MD	hassan.hosseini@aphp.fr	Paris / France	acquisition of Data	Kid Clot
ADRIEN	VILLAIN		MD	adrien.villain@aphp.fr	Paris / France	acquisition of Data	Kid Clot
CHANTAL	LAMY		MD	lamy.chantal@chu-amiens.fr	Amiens / France	acquisition of Data	Kid Clot
LOÏC	HERY		MD	hery.loic@chu-amiens.fr	Amiens / France	acquisition of Data	Kid Clot
CYRIL	CHIVOT		MD	chivot.cyril@chu-Amiens.fr	Amiens / France	acquisition of Data	Kid Clot
SOPHIE	GUEDEN		MD	sogueden@chu-angers.fr	Angers / France	acquisition of Data	Kid Clot
BENJAMIN	BOUAMRA		MD	benjamin.bouamra@wanadoo.fr	Besançon / France	acquisition of Data	Kid Clot
JOANNA	BELLEVILLE GOFFENEY		MD	jgoffeney@chu-besancon.fr	Besançon / France	acquisition of Data	Kid Clot
ALESSANDRA	BIONDI		MD PhD	biondi.alessandra@gmail.com	Besançon / France	acquisition of Data	Kid Clot
PAULINE	RENOU		MD	pauline.renou@chu-bordeaux.fr	Bordeaux / France	acquisition of Data	Kid Clot
MARIE	THIBAUD		MD	marie.thibaud@chu-bordeaux.fr	Bordeaux / France	acquisition of Data	Kid Clot
GAULTIER	MARNAT		MD	gaultier.marnat@chu-bordeaux.fr	Bordeaux / France	acquisition of Data	Kid Clot
NATHALIE	BACH		MD	bach-n@chu-caen.fr	Caen / France	acquisition of Data	Kid Clot

Supplemental Online Content: Nonauthor Collaborators
*First name, last name, and suffix (if applicable) are required and will appear in PubMed.

***First Name and Middle Initial(s)**	***Last Name**	***Suffix (eg, Jr, III)**	Academic Degrees	Institution	Location (city, state/province, country)	Role or Contribution, eg, chair, principal investigator	Group (if more than 1 Group listed in the byline) and/or Subgroup (eg, Steering Committee)
ANNA	FERRIER		MD	fdurif@chu-clermontferrand.fr	Clermont Ferrand / France	acquisition of Data	Kid Clot
GANAELLE	REMERAND		MD	gremerand@chu-clermontferrand.fr	Clermont Ferrand / France	acquisition of Data	Kid Clot
EMMANUEL	CHABERT		MD PhD	echabert@chu-clermontferrand.fr	Clermont Ferrand / France	acquisition of Data	Kid Clot
YANNICK	BÉJOT		MD PhD	yannick.bejot@chu-dijon.fr	Dijon / France	acquisition of Data	Kid Clot
OLIVIER	DETANTE		MD PhD	olivier.detante@univ-grenoble-alpes.fr; odetante@chu-grenoble.fr	Grenoble / France	acquisition of Data	Kid Clot
ELODIE	LAMETERY		MD	elametery@chu-grenoble.fr	Grenoble / France	acquisition of Data	Kid Clot
FLORENCE	TAHON		MD	ftahon@chu-grenoble.fr	Grenoble / France	acquisition of Data	Kid Clot
CHARLOTTE	CORDONNIER		MD PhD	charlotte.cordonnier@univ-lille.fr	Grenoble / France	acquisition of Data	Kid Clot
JORIOT	SYLVIE		MD	sylvie.joriot@chru-lille.fr	Lille / France	acquisition of Data	Kid Clot
KAZEMI	APOLLINE		MD	apolline.kazemi@chru-lille.fr	Lille / France	acquisition of Data	Kid Clot
CECILE	LAROCHE		MD	cecile.laroche@chu-limoges.fr	Limoges / France	acquisition of Data	Kid Clot
SUZANA	SALEME		MD	s.saleme@mac.com	Limoges / France	acquisition of Data	Kid Clot
LAURENT	DEREX		MD PhD	laurent.derex@chu-lyon.fr	Lyon / France	acquisition of Data	Kid Clot
MARYLINE	CARNEIRO		MD	maryline.carneiro@chu-lyon.fr	Lyon / France	acquisition of Data	Kid Clot
OMER	EKER		MD PhD	omer.eker@chu-lyon.fr	Lyon / France	acquisition of Data	Kid Clot
FREDERIQUE	AUDIC		MD	frederique.audic@ap-hm.fr	Marseille / France	acquisition of Data	Kid Clot
PHILIPPE	DORY LAUTREC		MD	philippe.dory-lautrec@ap-hm.fr	Marseille / France	acquisition of Data	Kid Clot
NADINE	GIRARD		MD PhD	nadine.girard@ap-hm.fr	Marseille / France	acquisition of Data	Kid Clot
CAROLINE	ARQUIZAN		MD	c-arquizan@chu-montpellier.fr	Montpellier / France	acquisition of Data	Kid Clot
PIERRE	MEYER		MD	p-meyer@chu-montpellier.fr	Montpellier / France	acquisition of Data	Kid Clot
SEBASTIEN	RICHARD		MD PhD	s.richard@chru-nancy.fr	Nancy / France	acquisition of Data	Kid Clot
CLAIRE	BILBAULT		MD	neuropediatrie.bilbault@gmail.com	Nancy / France	acquisition of Data	Kid Clot
HUBERT	DESAL		MD PhD	hubert.desal@chu-nantes.fr	Nantes / France	acquisition of Data	Kid Clot
ANNE	ROLLAND		MD	anne.rolland@chu-nantes.fr	Nantes / France	acquisition of Data	Kid Clot
BOURCIER	ROMAIN		MD PhD	romain.bourcier2@gmail.com	Nantes / France	acquisition of Data	Kid Clot

Supplemental Online Content: Nonauthor Collaborators
*First name, last name, and suffix (if applicable) are required and will appear in PubMed.

***First Name and Middle Initial(s)**	***Last Name**	***Suffix (eg, Jr, III)**	Academic Degrees	Institution	Location (city, state/province, country)	Role or Contribution, eg, chair, principal investigator	Group (if more than 1 Group listed in the byline) and/or Subgroup (eg, Steering Committee)
EMMANUELLE	GONDON		MD	gondon.e@pediatrie-chulenval-nice.fr	Nice / France	acquisition of Data	Kid Clot
JACQUES	SEDAT		MD	jsedat@yahoo.fr	Nice / France	acquisition of Data	Kid Clot
PASCAL	AUZOU		MD	pascal.auzou@chr-orleans.fr	Orléans / France	acquisition of Data	Kid Clot
CANAN	OZSANCAK		MD	canan.ozsancak@chr-orleans.fr	Orléans / France	acquisition of Data	Kid Clot
GUILLAUME	CAMI		MD	guillaume.cami@chr-orleans.fr	Orléans / France	acquisition of Data	Kid Clot
JEAN PHILIPPE	NEAU		MD PhD	jean-philippe.neau@chu-poitiers.fr	Poitier / France	acquisition of Data	Kid Clot
NICOLAS	RAYNAUD		MD	nico.raynaud2@gmail.com	Poitier / France	acquisition of Data	Kid Clot
STÉPHANE	VELASCO		MD	velascostephane@free.fr	Poitier / France	acquisition of Data	Kid Clot
STEPHANE	VANNIER		MD	stephane.vannier@chu-rennes.fr	Rennes / France	acquisition of Data	Kid Clot
LÉNA	DAMAJ		MD	lena.damaj@chu-rennes.fr	Rennes / France	acquisition of Data	Kid Clot
JEAN CHRISOPHE	FERRÉ		MD PhD	jean-christophe.ferre@chu-rennes.fr	Rennes / France	acquisition of Data	Kid Clot
FRANCOIS	EUGENE		MD	francois.eugene@chu-rennes.fr	Rennes / France	acquisition of Data	Kid Clot
AUDE	TRIQUENOT BAGAN		MD	aude.triquenot@chu-rouen.fr	Rouen / France	acquisition of Data	Kid Clot
CHRISANTHI	PAPAGIANNAKI		MD	chrisanthi.papa@gmail.com	Rouen / France	acquisition of Data	Kid Clot
VALÉRIE	WOLFF		MD PhD	valerie.wolff@chru-strasbourg.fr	Strasbourg / France	acquisition of Data	Kid Clot
ALEXANDRA	PEREZ		MD	alexandra.perez@chru-strasbourg.fr	Strasbourg / France	acquisition of Data	Kid Clot
RÉMY	BEAUJEUX		MD PhD	remy.beaujeux@chru-strasbourg.fr	Strasbourg / France	acquisition of Data	Kid Clot
EMMANUEL	CHEURET		MD	cheuret.e@chu-toulouse.fr	Toulouse / France	acquisition of Data	Kid Clot
JEAN	DARCOURT		MD	darcourt.j@chu-toulouse.fr	Toulouse / France	acquisition of Data	Kid Clot
KEVIN	JANOT		MD	kevin.janot@hotmail.com	Tours / France	acquisition of Data	Kid Clot
MAXIMILIEN	PERIVIER		MD	m.perivier@chu-tours.fr	Tours / France	acquisition of Data	Kid Clot
DENIS	HERBRETEAUX		MD PhD	herbreteau@med.univ-tours.fr	Tours / France	acquisition of Data	Kid Clot
